# Tick abundance: a one year study on the impact of flood events along the banks of the river Danube, Austria

**DOI:** 10.1007/s10493-017-0114-1

**Published:** 2017-03-01

**Authors:** Martin Weiler, Georg Gerhard Duscher, Monika Wetscher, Julia Walochnik

**Affiliations:** 1 Institute of Specific Prophylaxis and Tropical Medicine, Medical University of Vienna, Vienna, Austria; 2 Institute of Parasitology, University of Veterinary Medicine, Veterinaerplatz 1, 1210 Vienna, Austria; 3 CBRN-Defense School, Austrian Armed Forces, Korneuburg, Austria

**Keywords:** Ticks flooding, Barrier, Sedimentation

## Abstract

The abundance of questing ticks depends on various factors. In this study, the impact of a major flood event on tick abundance and activity was observed. Ticks were collected on a weekly basis in two approximately 2 km^2^ large floodplain areas on the inner and the outer bank of the river Danube north of Vienna, Austria. In 2013 before a 200 year flood event, an average of 55 ticks per hour was collected in the area on the outer bank and 21 ticks per hour in the area on the inner bank. After the flood event the tick activity was massively reduced, with 12 ticks per hour on the outer bank and 1.1 ticks per hour on the inner bank. The most distinctive factor between the two areas was the level of sediment after the flooding, with almost no sediment in the outer bank, whereas on the inner bank the average height of sediment was 270 mm. Our data indicate the residual sediment has a greater impact on tick abundance and activity than the flooding itself. Besides the direct effect of ticks being buried under the sediment, there may be important indirect effects of the sediment on the habitat of the ticks and/or the host animals. We assume that this is the reason for the generally significantly lower numbers of questing ticks in this area on the inner bank of the Danube in this region, with periodical flood events.

## Introduction

Ticks are the most important vectors of pathogens in Central Europe, including Austria. The most important tick species in Austria is *Ixodes ricinus*, which is widely distributed with high densities below 1000 m. Thus, the distribution and abundance of these ticks is a major health issue. Numerous studies have been performed to obtain reliable data on the abundance of ticks and their pathogens and to define temperature and humidity ranges to predict tick occurrence and activity (Perret et al. 2000; Gray 2008; Knap et al. 2009). Dramatic events, such as a massive flooding, might have an impact on the geographical distribution and abundance of ticks. Burning as well as mowing of the vegetation is known to cause a decrease of questing adult ticks for up to 12 months (Wilson 1986). Although ticks are able to survive for weeks under water under laboratory conditions (Dautel et al. 2011) and it is assumed that at least 3 weeks of submersion in water are necessary to kill unfed ticks in the environment, most of the tick eggs die during periodic flood events of 1–2 weeks (Koch 1986). During shorter flooding events at least the hatchability of the eggs will decrease over time, differing however significantly between tick genera and species (Adejinmi 2011). The comparably long survival of ixodid ticks under water is facilitated by plastron respiration, reduced metabolism and possible anaerobic respiration (Fielden et al. 2011). Moreover, there are indirect effects of flood events on the tick population, such as killing or expelling the hosts, dramatic changes in the vegetation or significant sedimentation. Differences in the leaf litter and vegetation can have an effect on the presence and on the survival of ticks, as well as on the risk of disease transfer (van Overbeek et al. 2008).

The aim of this study was to show the effect of a major flooding on the tick abundance. Therefore we investigated two floodplain areas along the river Danube, similar in geology, vegetation, geographical position and fauna, but one on the inner bank and one on the outer bank in the river bend north of Vienna. Ticks were collected on a weekly basis before and after the a 200 year flood event.

## Materials and methods

**Fig. 1:**
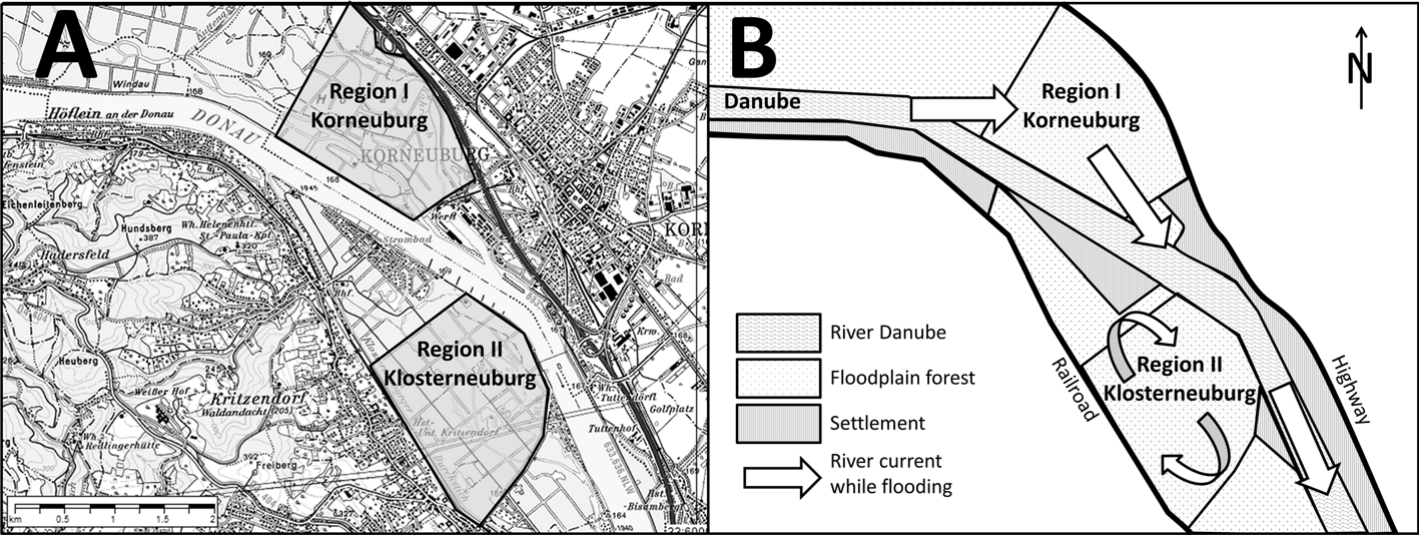
**A** The sampling sites, north and south of the Danube, approximately 5 km north of Vienna, Lower Austria, Austria. **B** Currents in the river at region I and II. The *arrows* indicate the current in the river. During a flooding the stream runs straight through region I, whereas there is circulation in II

The study was performed in two comparable areas, which are separated by the river Danube, in Lower Austria approximately 5 and 8 km north of Vienna. The first study site comprised a 1.6 km^2^ large area on the outer (northern) bank of the Danube (48°18′N, 16°19′E, average altitude 165 m a.s.l.) belonging to the municipality Korneuburg. The second study site was a 2.3 km^2^ large area on the inner bank of the Danube belonging to the municipality Klosterneuburg, south of the Danube (48°18′N, 16°19′E, average altitude 165 m a.s.l.) (Fig. 1a). the vegetation was analyzed at 10 random positions in an approximate distance of 50 m, in both areas. At the same positions the sedimentation levels were recorded 45 days after the flood event. Both areas are covered by floodplain forest with mainly, deciduous trees and shrubs. Trees are cut regularly in both areas. Soil samples were taken with an impact drill and a cube cut. The ground layer was lifted and the height of the top sediment level was measured. Both areas are habitats for a wide variety of animals, including ungulates, canids and rodents, which may act as hosts for ticks. The water level on the sites was measured after the water had declined by measuring the span between the ground level and a visible mark caused by the water surface level on straighten upright tree standing on flat ground.

Ticks were collected in both regions by using the flagging method with a standardized flag of one square meter, once a week, from April 2013 until December 2013. With this method, only active questing ticks can be caught, which does not represent the absolute number of ticks in an area, but it allows a comparison of relative tick abundance. Sampling was performed for ½–1 h per region depending on tick density by one and the same collector to achieve comparable collection results. With the collection time and the tick numbers the rate of collected ticks per hour was calculated, in order to be able to compare the sampling results. Temperature and humidity on the ground level were measured with a portable digital temp/humidity meter (Kestrel 4000 NV, Pocket Weather Tracker) at the beginning of the collection process. The ticks were cooled at 4 °C for up to 2 weeks, due to logistical reasons, until they were stored in a deep-freezer at −80 °C. Species and instars were identified morphologically, using identification keys (Hillyard 1996; Estrada-Peña et al. 2004). Larvae were excluded from the study. The calculated reduction is based on the predominant *I. ricinus* species.

## Results

**Fig. 2:**
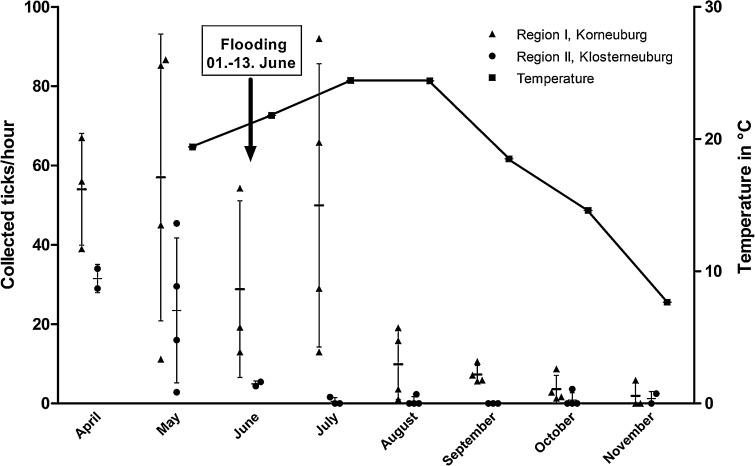
Number of ticks collected per hour in region I (Korneuburg) and region II (Klosterneuburg) and the respective average temperature including monthly mean value and standard deviation. Every circle or triangle represents one single collection

**Fig. 3:**
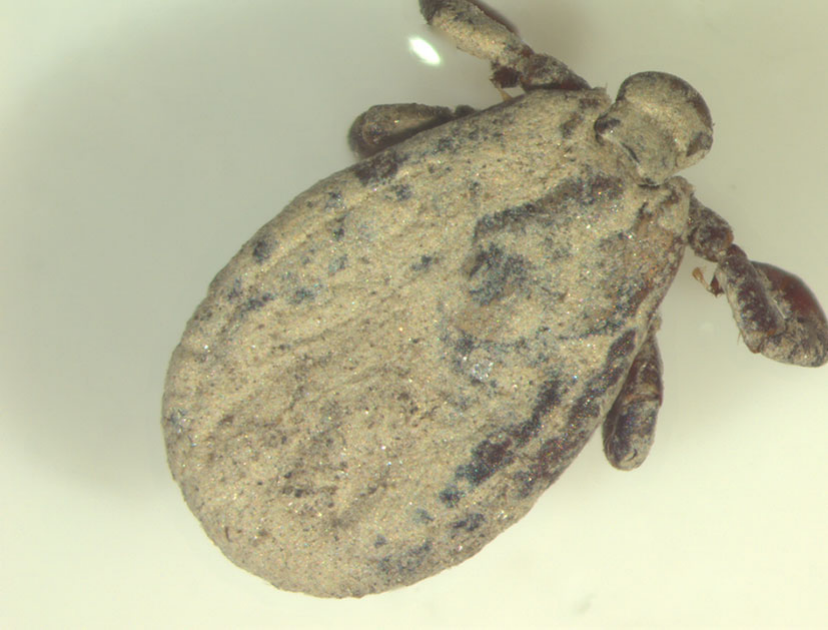
*Dermacentor reticulatus* covered with sand, flagged 4 months after the flood event

A total of 926 ticks were collected from April to December 2013, i.e. 702 in the area on the outer bank (region I) and 224 in the area on the inner bank (region II). Three species were found: 90.1% *I. ricinus*, 6.4% *Haemaphysalis concinna* and 3.5% *Dermacentor reticulatus*. The highest numbers of ticks per month were collected in the 2 months before the flood (Fig. 2). The total number of ticks collected in the 6 months after the flood decreased to 294 on the outer bank and to 31 on the inner bank. In the outer bank area, on average 55 ticks per hour were collected before the flooding and 12 ticks per hour after the flooding, meaning a 4.6 fold decrease. An even more pronounced decrease of collected ticks per hour was seen on the inner bank, where the collection rate decreased 20-fold from 21 to 1.1 ticks per hour (Fig. 2). Focusing on the collection results from June, directly after the flood, the collection rate on the outer bank decreased from 56 ticks per hour in May to 29 ticks per hour (1.9 fold reduction). On the inner bank, the collection rate dropped from 22 to 5 collected ticks per hour (4.4 fold reduction). On the outer bank, live ticks covered with sand were still found 4 month after the flood (Fig. 3).

**Fig. 4:**
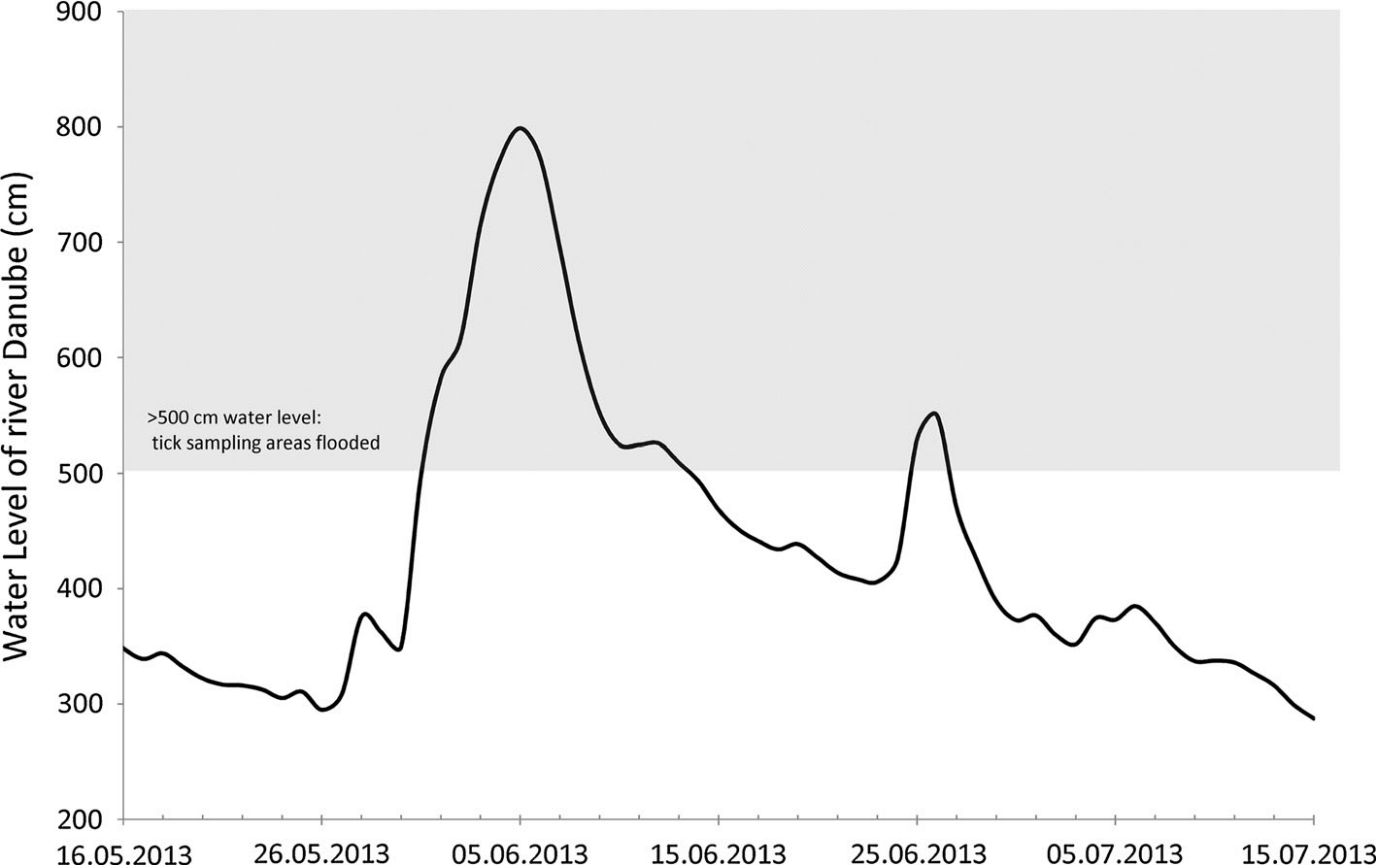
Water level of the river Danube in the tick sampling region during the flooding 2013

The maximum water level of the river Danube reached 809 cm on June 5th, 2013 at 9:00 pm. This flood represented a 200 year event (“Water levels in Lower Austria, Department of Hydrology and Geo-Information, Office of the Provincial Goverment of Niederösterreich,” n.d.). On the tick sampling sites, a water level of 270 cm was reached on the outer bank and of 310 cm above ground on the inner bank. The daily average water level was constantly above 500 cm over a period of 2 weeks (01.06.–13.06.2013), implicating totally flooded tick sampling areas (Fig. 4). The previous major floods were recorded on June 23rd, 2009: 699 cm, September 7th, 2007: 698 cm and August 15th, 2002: 789 cm. Smaller floodings with a water level above 500 cm for one or a few days occur several times every year: e.g. four times in 2013 and two times in 2015 (Data provided by Department of Hydrology and Geo-Information, Office of the Provincial Goverment of Niederösterreich and Donau River Information Services, viadonau GesmbH).

Both tick sampling sites were on floodplain ground in direct proximity to the Danube, but as the Danube in this region bends slightly from west to south, the currents differ markedly between the north shore (outer bank, fast flow) and south shore (inner bank, slow flow). Additionally, the water flow along the inner bank is blocked by settlements (Fig. 1b). This resulted in significantly different levels of sedimentation on the two tick sampling sites: the calculated mean of sedimentation was 0.4 mm on the outer bank site and 270 mm on the inner bank site. The leaf litter was visible with bare eye on the outer bank, while on the inner bank it was only visible after the sediment had been removed.

The vegetation differed in composition up to 1 m above ground level after the flood: on the outer bank site, the composition was 70% *Rubus caesius*, 10% juvenile *Acer negundo*, each with 5% *Calamagrostis epigejos*, *Caltha palustris*, *Impatiens glandulifera* and *Carex sylvatica; Viola reichenbachiana* and *Carex remota* were also seen sporadically, while on the inner bank site the vegetation was composed of 70% *Urtica dioica* and 30% *R. caesius,* with a sporadic distribution of *C. epigejos, C. palustris,* and *I. glandulifera*.

## Discussion

It was rather startling to see that tick activities were so different in two similar and close-by habitats, only divided by the river Danube. And this difference became even more pronounced after the flood that occurred in the sampling area. Both areas are regularly flooded completely for a period of several days every few years. Short time floodings of one or two days occur even more often. Both sampling areas are located at almost the same altitude. Both sites were flooded with at least 270 cm of water above ground level during the event in June 2013. A more accurate recording of the water level was difficult because of the uneven floodplain land structure. However, the comparably small difference of the level of the water column (~40 cm) cannot be expected to have a significant impact on tick survival. But 2 weeks after the water had declined, significant differences in the sedimentation of river sand were found, while the alluvium was only a few millimeters on the outer bank, it was 27 cm on the inner bank. Consequently, there was a drastic change in the vegetation in this area. Here, *U. dioica* (common nettle) covered almost the entire surface, while *U. dioica* was not found on the outer bank. This plant is a ruderal plant and prefers moist ground with a high level of nitrogen, but is also known to survive and rapidly re-establish after fire (Taylor 2009). The effect of the alluvium was also reflected by the number of collected ticks. On the outer bank, with low levels of alluvium, only a minor reduction of tick density was recorded directly after the flood in June. The density increased in July, and then slowly decreased during the rest of the year. The increase in July suggests that the effect of the flood in this area was limited to direct effects of the water. The relatively low tick densities during the summer months reflect the normal effect of the hot period that can be observed every year. In autumn, a second peak of tick activity is normally observed (Egyed et al. 2012; Dantas-Torres and Otranto 2013). This peak was missing after the flood in the area of the outer bank, and visible but reduced in the area of the inner bank. On the inner bank sampling site, the number of collected ticks per hour was fourfold reduced directly after the flood and remained on a very low level for the rest of the year, with no increased activity in July. As major flood events occur on a regular basis in this region and as we could show that the area on the inner bank is significantly more affected by flood events, we conclude that there is an overall lower tick activity in this area. This conclusion is corroborated by the fact that also before the flood, i.e. 4 years after the previous flood, significantly lower tick activity was observed. We further conclude that not the direct effect, the water, has a major impact on tick activity, but rather the indirect, long-term effects of a flood event. The critical factor is the level of alluvium, while moderate alluvium, even if entirely covering the individual ticks, will not kill the ticks, as seen for *D. reticulatus* even 4 months after the flood in the current study, extensive alluvium, as observed in the area on the inner bank, permanently buries the ticks, with a major and long term impact on tick abundance. Even if individual ticks manage to escape the sediment, there is no leaf litter left for the ticks to escape heat and desiccation. Schulze and colleagues showed that the removal of leaf litter in spring and in the early summer can strongly reduce the prevalence of nymphs and larvae (Schulze et al. 1995). The effect of the flood event on the various blood hosts is not easy to evaluate. While many might have been able to escape, several e.g. of the wild ungulates drowned, reducing the number of potential hosts. However, the population of the wild ungulates is not assumed to be the bottle neck for tick development, because very few individuals can sustain the tick population (Ostfeld et al. 2006). Even though roe deer can also act as blood hosts for the immature stages of ticks (Kiffner et al. 2011), the survival of the rodent populations, being the main hosts for larvae and nymphs, are certainly more critical for overall tick abundance. Although we have no data on the rodent density before and after the flood, it is known that the rodent population is decreased after flooding (Zhang et al. 2007). The water level and the duration of the flood event were rather similar in both areas investigated, thus we assume that also the impact of the water on the rodent population was comparable. But also here, the level of alluvium probably is critical, as rodents will be trapped and underground rodent lairs will be clogged permanently and not only drowned during the flooding.

In summary, this study demonstrates the dramatic effects of a flooding event on tick abundance, whereby the major impact was caused by heavy sedimentation, either directly or indirectly by affecting the availability of potential hosts.
